# Job Satisfaction, Leadership Behaviors, and Turnover Intention Among Nurses in Saudi Arabia: A Mediation Analysis

**DOI:** 10.1155/jonm/4705174

**Published:** 2026-09-22

**Authors:** Naji Alqahtani, Afaf M. Albylwi

**Affiliations:** ^1^ Nursing Administration and Education Department, Collage of Nursing, King Saud University, Riyadh 11421, Saudi Arabia, ksu.edu.sa; ^2^ College of Nursing, King Saud University, Riyadh 11421, Saudi Arabia, ksu.edu.sa

**Keywords:** behavior, job satisfaction, leadership, nursing, retention strategies, Saudi Arabia, turnover intention

## Abstract

**Aim:**

To examine the associations among nurses’ perceived leadership behavior, job satisfaction, and turnover intention in Saudi Arabia and to determine whether perceived leadership behavior mediates the relationship between job satisfaction and turnover intention.

**Background:**

Nurses’ turnover intention remains a major challenge for healthcare systems worldwide. High turnover contributes to nursing shortages, negatively affects patient outcomes, and increases healthcare costs. Although job satisfaction is a well‐established predictor of turnover intention, limited research has explored the potential mediating role of perceived leadership behavior in this relationship.

**Methods:**

An advanced correlational design was used. Data were collected via online questionnaires completed by nurses working in selected hospitals. Leadership behavior, job satisfaction, and turnover intention were measured using the Leader Behavior Description Questionnaire, Job Satisfaction Survey, and turnover intention scale, respectively. Descriptive statistics, Pearson’s correlation, and multiple linear regression were used. Mediation was tested using Hayes’ PROCESS Macro, Version, 5 Model 4, with bootstrap procedures. Turnover intention was designated as the primary outcome because it is the most proximal and modifiable behavioral predictor of actual turnover.

**Results:**

Nurses reported moderate levels of job satisfaction, turnover intention, and perceived leadership behavior. Turnover intention was moderately and negatively correlated with perceived leadership behavior (*r* = −0.481, *p* < 0.001). Perceived leadership behavior partially mediated the relationship between job satisfaction and turnover intention (*B* = −0.036, 95% CI [−0.066, −0.012]) and accounted for 30.3% of the total effect.

**Conclusion:**

Lower job satisfaction was associated with higher turnover intention. Perceived leadership behavior statistically mediated part of this relationship. Supportive, well‐structured leadership independently increases and reduces turnover intentions. Therefore, strengthening leadership behavior is a practical approach for improving nurse retention.

**Implications for Nursing Management:**

Implementing effective leadership strategies and leadership development programs may improve nurses’ job satisfaction and reduce their intention to leave.

## 1. Introduction

Nurse turnover intention is widely recognized as a critical challenge affecting healthcare systems worldwide. The World Health Organization projected a global shortfall of approximately 5.7 million nurses by 2030 [1]. The International Council of Nurses has also reported intention‐to‐leave rates of 20% or higher and annual hospital turnover rates of at least 10% in many settings [2]. Compared with professionals in many other fields, nurses report higher intentions to leave their jobs [3]. High turnover contributes to workforce shortages, negatively affecting patient outcomes, and increases healthcare costs [4]. As turnover intention is considered the most proximal and predictive indicator of turnover, it is commonly used as an early indicator in workforce planning [5]. Identifying the factors associated with nurses’ intentions to leave is therefore important to mitigate voluntary turnover and its organizational consequences [6].

Job satisfaction is a major predictor of nurses’ turnover intention among nurses. Low job satisfaction is consistently associated with increased intention to leave among nurses [7]. It has also been linked to poor job performance, low productivity, and high staff turnover, whereas nurses with higher job satisfaction are more likely to show positive outcomes and reduced turnover [8, 9]. Although previous studies have reported a direct negative association between job satisfaction and turnover intention, limited attention has been given to the factors that may help explain this relationship [10].

Several factors influence both job satisfaction and turnover intention, among which leadership behavior is considered a key determinant [11]. Early leadership research primarily focused on leaders’ personality traits, whereas later studies emphasized observable leadership behavior as a more influential factor [12]. Path–goal theory identifies directive, supportive, participative, and achievement‐oriented leadership behaviors as factors associated with employee job satisfaction [13]. These behaviors have also been associated with both staff productivity and job satisfaction [14].

Leadership behavior refers to the observable leadership behaviors as factors demonstrated by individuals in leadership positions [14]. These behaviors are influenced by individual characteristics, organizational values, and situational demands [15]. Different situations may require different leadership styles. Therefore, effective leaders should recognize employees’ emotional needs, understand their motivations, and support them in achieving organizational goals. Leadership behavior is among the most consistent organizational factors associated with job satisfaction and turnover intention in nursing [16]. Unlike other demographic and structural factors, leadership behavior is potentially modifiable through targeted training and management practices, which makes it a possible practical focus for nurse‐retention strategies.

In Saudi Arabia, where the majority of the nursing service relies on expatriate nurses, turnover rates in large hospitals exceed 20% [17]. This reliance on foreign workforce makes it important to understand the factors associated with nurses’ intention to leave. In addition, restricted leadership opportunities and current nursing management practices have also been linked to job dissatisfaction and turnover in the country [17]. Therefore, this study highlights the importance of perceived leadership behavior to nurse retention in the country.

Although evidence supports the correlations among leadership behavior, job satisfaction, and turnover intention, little is known about the mediating role of perceived leadership behavior in explaining how job satisfaction influences turnover intention among nurses, particularly in the Saudi Arabian healthcare context. Accordingly, this study examined the mediating role of nurses’ perceived leadership behavior in job satisfaction and turnover intention relationship.

## 2. Materials and Methods

### 2.1. Research Design

An advanced correlational design utilizing multiple regression and mediation modeling via bootstrapping was used in this study.

### 2.2. Research Setting

The study was conducted in three hospitals in Riyadh, Saudi Arabia, including teaching, public, and private healthcare institutions.

### 2.3. Population and Sample

The target population consisted of registered nurses employed across the three selected hospitals. Participants were recruited using a convenience sampling technique. Eligible participants were nurses who provided direct patient care, had worked as a nurse at their current institution for at least 6 months, and voluntarily agreed to participate. Nurses holding managerial/leadership positions, such as head nurses, or charge nurses were excluded.

#### 2.3.1. Sample Size Calculation

G∗Power software Version 3.1 was used to calculate the minimum sample size required for the statistical analysis. At a significance level of 0.05, an effect size of 0.15, a power of 0.8, and eight independent variables, the minimum sample size required to perform a multiple regression analysis was 109 participants. For the mediation modeling, the required sample size provides adequate statistical power [18]. Of the 600 invited participants, 140 completed the questionnaires, corresponding to a response rate of 23.3%.

### 2.4. Measurements

Data were collected using structured questionnaires. Four instruments were used in this study.

#### 2.4.1. Demographic and Work‐Related Characteristics

The demographic and work‐related variables included age, gender, nationality, educational level, years of experience, and working hospital.

#### 2.4.2. Job Satisfaction

Job satisfaction was assessed using the Job Satisfaction Survey (JSS) [19]. The JSS contains 36 items distributed across nine dimensions: pay, promotion, supervision, fringe benefits, contingent rewards, operating conditions, coworkers, nature of work, and communication. Each dimension consists of four items. Items are rated on a six‐point Likert scale ranging from 1 (*disagree very much*) to 6 (*agree very much*). A sample item from the scale is “Communications seem good within the organization.” The total score was the sum of the 36 items, which ranged from 36 to 216 (reported as a mean of 1–6). Each of the nine subscale scores is based on four items and ranges from 4 to 24 (reported as a mean of 1–6). Higher scores on the scale indicate greater job satisfaction. The JSS has shown strong psychometric properties, internal consistency (Cronbach’s *α* = 0.91), and established validity [19].

#### 2.4.3. Leadership Behavior

Perceived leadership behavior was measured using the 30‐item short form of the Leader Behavior Description Questionnaire (LBDQ) [20]. The questionnaire provides an approach whereby staff describe the observable behaviors of their leaders in an organization. It evaluates two dimensions of leadership behavior: consideration, which reflects interpersonal support and warmth, and initiating structure, which reflects task organization and role clarity. The questionnaire contains 15 items assigned to each dimension. A sample initiating structure item is “He lets group members know what expected of them.” A sample consideration item is “He is willing to make changes.” Items are measured on a five‐point Likert scale ranging from 0 (*never*) to 4 (*always*). The overall possible score for each dimension is 60 (reported as a mean of 0–4), with higher scores reflecting more positive perceptions of leadership behavior in each dimension. The total score was calculated by summing the scores of the two dimensions, resulting in a possible total score of 120 (reported as a mean of 0–4). The LBDQ has demonstrated strong psychometric properties. Reliability was estimated using the split‐half method and was reported to be 0.83 for the initiating structure and 0.92 for the consideration behavior scores [20].

#### 2.4.4. Turnover Intention

Turnover intention was assessed using the six‐item turnover intention scale (TIS‐6) [21]. The scale consists of six items rated on a five‐point Likert scale. A sample item is “How often have you considered leaving your job?” Response options ranged from 1 (*low turnover intention*) to 5 (*high turnover intention*). Higher scores indicated a greater intention to leave the organization. The TIS‐6 demonstrated acceptable validity and reliability, with a reported Cronbach’s *α* of 0.80 [21].

### 2.5. Data Collection Procedure

Following ethics approval, data were gathered electronically utilizing a secure online platform between November 1, 2025, and December 20, 2025. Participation reflected the availability of nurses in each setting. A self‐administered questionnaire was organized into four sections: demographic and work‐related characteristics, the JSS, the LBDQ, and the TIS‐6. The questionnaire took approximately 20 min to complete.

An invitation email containing a link to the survey was distributed to eligible participants using centralized institutional hospital communication systems. Only eligible nurses were invited to participate. Permission to distribute the questionnaire was obtained through the nursing departments. Before accessing the questionnaire, participants were presented with an information page describing the study’s purpose, the voluntary nature of participation, and their right to withdraw at any time without consequence. Participants were also assured that their responses would remain anonymous and confidential. Electronic informed consent was obtained from all participants prior to data collection.

To enhance the response rate, two reminder emails were sent at 2‐week intervals following the initial invitation. Submitted questionnaires were screened for completeness before analysis.

### 2.6. Ethical Considerations

Ethical approval was obtained from the institutional review board (IRB). Participants were assured that their information would remain confidential and their privacy would be protected throughout the study. Informed consent was obtained before participation, and participants were fully informed about the purpose of the research. All collected data were anonymized and securely stored to ensure data protection.

### 2.7. Data Analysis Plan

All analyses were performed using a licensed copy of the IBM SPSS Statistics software, Version 31.0 (IBM Corp., Armonk, NY, USA). The distribution of each continuous variable was examined using skewness and kurtosis, the Shapiro–Wilk test, histograms, and Q–Q plots. The main study variables were approximately normally distributed (skewness and kurtosis within ±2) and are presented as mean ± SD and analyzed with parametric tests; age and years of experience were mildly positively skewed and are additionally presented as median (interquartile range). Categorical variables were shown as frequencies and percentages.

For the bivariate analyses, the participants’ turnover intention scores were divided at the midpoint into lower and higher turnover intention groups. Then, the sample characteristics and the study variables were then compared between the two groups. Normally distributed variables were compared using an independent sample *t*‐test. Age and years of experience were compared using the Mann–Whitney *U* test. Categorical variables were analyzed using Pearson’s chi‐squared test.

Turnover intention was retained as a continuous variable for the correlation, regression, and mediation analyses. Pearson’s correlation coefficient was calculated to examine the relationship between job satisfaction, leadership behavior, and turnover intention. Multiple linear regression analysis was performed to examine the effects of job satisfaction and leadership behavior on turnover intention while controlling for demographic variables. Before interpretation, regression assumptions were assessed and met: linearity and homoscedasticity, normality of residuals (normal P–P plot; standardized residuals ranged from −2.70 to 2.23), independence of errors (Durbin–Watson = 1.84), and absence of multicollinearity (all tolerance ≥ 0.26, all variance inflation factors ≤ 3.78). Continuous predictors were approximately normally distributed.

Finally, the hypothesized role of leadership behavior in the relationship between job satisfaction and turnover intention was tested using Hayes’ PROCESS Macro, Version 5, Model 4, with bootstrapping procedures, as recommended by Hayes [22]. Bootstrap resampling was used because the sampling distribution of the indirect effects was non‐normal. The indirect effect was the product of the two regression coefficients (Paths a and b). The distribution of this product is asymmetric even when the variables are normally distributed. Bootstrapping provides a more accurate and powerful inference for indirect effects [23, 24]. The indirect medicating effect was considered statistically significant when the bootstrap confidence interval did not include a zero.

## 3. Results

### 3.1. Demographic Characteristics and Study Variables

The study comprised 140 nurses. The participants’ demographic characteristics and comparisons by turnover intention groups are presented in Table 1. The median participant age was 32 years (IQR = 28–38; range 23–63). The median duration of professional nursing experience was 7 years (IQR = 4–13; range 1–36). Most participants were female (84.4%) and expatriates nurses (90.7%), and more than two‐thirds of the sample (70.7%) held a bachelor’s degree. The largest proportion of nurses worked in a private hospital (44.3%), followed by a teaching hospital (37.9%) and a public hospital (17.9%).The mean overall job satisfaction score was 3.65 (SD = 0.59; range 1–6). Among its subscales, nurses were most satisfied with the nature of their work (*M* = 4.16), supervision (*M* = 4.08), and coworkers (*M* = 4.02), and they were least satisfied with operating conditions (*M* = 3.11) and compensation (*M* = 3.29). Perceived leadership behavior had a mean of 2.63 (SD = 0.70; range 0–4), with the initiating structure subscale rated higher mean score (*M* = 2.79, SD = 0.75) than the consideration subscale (*M* = 2.47, SD = 0.74). The mean turnover intention score indicated a moderate level of turnover intention (*M* = 2.97, SD = 0.81; range 1–5).

**TABLE 1:** Demographic characteristics and study variables by turnover‐intention group (*N* = 140).

Variable	Total (*N* = 140)	Lower TI (*n* = 62)	Higher TI (*n* = 78)	Test statistic	*p*
*Demographic characteristics*					
Age, years, median (IQR) M ± SD	32 (28–38) 33.8 ± 7.66	33 (29–40)	31 (28–37)	*U* = 2055.0	0.168
Years of experience, median (IQR) M ± SD	7 (4–13) 8.98 ± 6.75	8 (4–14)	6 (4–11)	*U* = 2146.5	0.254
Gender, *n* (%)				*χ* ^2^ = 8.56	0.003
Female	118 (84.3)	46 (74.2)	72 (92.3)		
Male	22 (15.7)	16 (25.8)	6 (7.7)		
Nationality, *n* (%)				*χ* ^2^ = 0.02	0.887
Non‐Saudi	127 (90.7)	56 (90.3)	71 (91.0)		
Saudi	13 (9.3)	6 (9.7)	7 (9.0)		
Educational level, *n* (%)				*χ* ^2^ = 1.13	0.288
Bachelor’s (BSN)	99 (70.7)	41 (66.1)	58 (74.4)		
Diploma	41 (29.3)	21 (33.9)	20 (25.6)		
Working hospital, *n* (%)				*χ* ^2^ = 7.35	0.025
Teaching	53 (37.9)	27 (43.5)	26 (33.3)		
Public	25 (17.9)	5 (8.1)	20 (25.6)		
Private	62 (44.3)	30 (48.4)	32 (41.0)		

*Study variables (item mean, M ± SD)*					
Job satisfaction	3.65 ± 0.59	3.93 ± 0.60	3.42 ± 0.47	*t* = 5.57	< 0.001
Compensation	3.29 ± 0.98				
Promotion	3.61 ± 0.80				
Supervision	4.08 ± 1.08				
Fringe benefits	3.57 ± 0.80				
Contingent rewards	3.69 ± 0.59				
Operating conditions	3.11 ± 0.64				
Coworkers	4.02 ± 0.90				
Nature of work	4.16 ± 0.97				
Communication	3.87 ± 0.98				
Leadership behavior	2.63 ± 0.70	2.93 ± 0.59	2.40 ± 0.69	*t* = 4.81	< 0.001
Consideration	2.47 ± 0.74				
Initiating structure	2.79 ± 0.75				

*Correlations among study variables*					
Variable	M	SD	1	2	3
1. Job satisfaction	3.65	0.59	—		
2. Leadership behavior	2.63	0.70	0.589^∗∗∗^	—	
3. Turnover intention	2.97	0.81	−0.520^∗∗∗^	−0.481^∗∗∗^	—

*Note:* Percentages are within the turnover‐intention group (column %). The independent‐samples *t*‐test or Mann–Whitney *U* test were used for continuous variables, and categorical variables were compared using Pearson’s chi‐squares test. Pearson’s r correlation test was used for the study variables (continuous variables).

^∗∗∗^ *p* < 0.001.

Comparison between turnover intention groups showed no statistically significant differences in age, years of experience, nationality, or educational level between nurses with lower and higher turnover intention (all *p* > 0.05). However, gender was statistically associated with turnover intention (*χ* ^2^ = 8.56, *p* = 0.003). Female nurses accounted for 92.3% of the high‐turnover‐intention group than in the low‐turnover‐intention group (74.2%). Similarly, working hospital was associated with turnover intention (*χ* ^2^ = 7.35, *p* = 0.025). More public hospital nurses (25.6%) were in the higher‐turnover‐intention group than in the in lower‐turnover‐intention group (8.1%), whereas the teaching and private hospitals showed no significant differences. Conversely, nurses with lower turnover intention had significantly higher job satisfaction than those with higher turnover intention (*t* = 5.57, *p* ≤ 0.001). They also reported more positive perceived leadership behavior than those in the higher‐turnover‐intention group (*t* = 4.81, *p* = < 0.001).

### 3.2. Relationship Analysis

As shown in Table 1, job satisfaction was moderately and positively correlated with perceived leadership behavior (*r* = 0.589, *p* < 0.001). In contrast, both job satisfaction and perceived leadership behavior were negatively correlated with turnover intention (*r* = −0.520, *p* < 0.001; *r* = −0.481, *p* < 0.001, respectively).

### 3.3. Multiple Linear Regression Analysis

Multiple regression analysis (Table 2) showed that job satisfaction (β = −0.372, *p* < 0.001) and leadership behavior (β = −0.216, *p* < 0.001) significantly predicted turnover intention. Together, these variables accounted for 37.1% of the variance in turnover intention (*R* ^2^ = 0.371, *p* < 0.001) controlling for demographic variables. Among the demographic variables, only gender was significantly associated with turnover intention (β = −0.108, *p* < 0.05).

**TABLE 2:** Multiple linear regression analysis predicting turnover intention.

Variables	*B*	SE	95% CI	*β*	*t*	*p*
Age	−0.041	0.084	[−0.207, 0.125]	−0.066	−0.488	0.626
Gender (female)^†^	−2.387	1.083	[−4.529, −0.245]	−0.183	−2.205	0.029
Nationality (non‐Saudi)^†^	1.129	1.380	[−1.602, 3.860]	0.069	0.818	0.415
Education (bachelor’s) ^†^	−0.675	0.905	[−2.466, 1.116]	−0.064	−0.746	0.457
Years of experience	0.017	0.099	[−0.180, 0.214]	0.022	0.171	0.865
Hospital: public^†^	1.389	1.045	[−0.679, 3.457]	0.112	1.329	0.186
Hospital: private^†^	1.021	0.838	[−0.638, 2.680]	0.107	1.218	0.225
Job satisfaction	−0.084	0.020	[−0.124, −0.044]	−0.372	−4.134	< 0.001
Leadership behavior	−0.049	0.021	[−0.091, −0.007]	−0.216	−2.303	0.023

*Note:* B, unstandardized coefficient; β, standardized coefficient. Model: *R* ^2^ = 0.371, *F* (9, 129) = 8.47, *p* < .001, regression diagnostics confirmed the model assumptions: Durbin–Watson = 1.84, all VIF ≤ 3.78.

Abbreviation: CI, confidence interval.

^†^Reference category.

### 3.4. Mediating Role of Leadership Behavior

**FIGURE 1:**
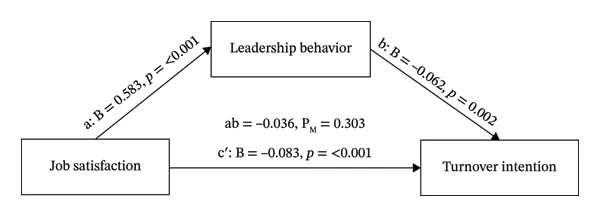
The mediating role of leadership behavior on the relationship between job satisfaction and turnover intention.

Table 3 and Figure 1 present the mediating effect of perceived leadership behavior on the relationship between job satisfaction and turnover intention. Job satisfaction had a significant direct effect on turnover intention (*B* = −0.083, 95% CI [−0.123, −0.044], *p* < 0.001). In addition, job satisfaction significantly predicted perceived leadership behavior (*B* = 0.583, *p* < 0.001), and perceived leadership behavior significantly predicted turnover intention (*B* = −0.062, *p* < 0.01). The indirect effect of job satisfaction on turnover intention through perceived leadership behavior was significant (*B* = −0.036, 95% CI [−0.066,−0.012]), indicating partial mediation. The proportion of mediation (P_M_), the ratio of the indirect effect to the total effect, was 0.303. This finding indicated that perceived leadership behavior mediated 30.3% of the effect of job satisfaction on turnover intention.

**TABLE 3:** Results of mediation analysis of leadership behavior using bootstrapping.

Path	*B*	SE	*t*	*p*	95% CI
a: Job satisfaction ⟶ leadership behavior	0.583	0.068	8.563	< 0.001	[0.448, 0.717]
b: Leadership behavior ⟶ turnover intention	−0.062	0.020	−3.067	0.002	[−0.103, −0.022]
c′: Job satisfaction ⟶ turnover intention (direct)	−0.083	0.020	−4.142	< 0.001	[−0.123, −0.044]
c: Job satisfaction ⟶ turnover intention (total)	−0.119	0.017	−7.146	< 0.001	[−0.153, −0.087]

**Bootstrapped indirect effect**					

**Indirect effect**	**B**	**Boot SE**	**95% bootstrap CI**	**Proportion mediated**	

ab: Job satisfaction ⟶ leadership behavior ⟶ turnover intention	−0.036	0.014	[−0.066, −0.012]	0.303 (30.3%)	

*Note:* Bootstrapping was performed with 5000 resamples. B, B coefficient.

Abbreviations: CI, confidence interval; SE, standard error.

## 4. Discussion

This study examined the associations among perceived leadership behavior, job satisfaction, and turnover intention among nurses in Saudi Arabia. The findings indicated that nurses perceived their leaders as demonstrating high initiating structure and moderate consideration. Nurses reported moderate job satisfaction and turnover intention. These findings highlight the influence of leadership practices on nurses’ workplace experiences and retention.

A higher initiating structure score reflects leaders providing clear task definitions, setting expectations, organizing workflows, and structuring clinical processes. According to House and Mitchell (1975), initiating structural behaviors promote role clarity and predictable work environments, which may enhance employee performance and direction [13]. Such structured leadership may be valuable in acute healthcare settings, where reducing ambiguity is essential for effective clinical practice. This may help explain why nurses value initiating structural behaviors by nursing leadership. In addition, it may reflect the organizational reality in Saudi health settings that operate using hierarchical and protocol management systems.

Consideration behavior reflects leaders’ friendliness, respect, and concern for staff well‐being. Increased job satisfaction, reduced burnout, and improved staff retention have been linked to relationship‐oriented leadership, including supportive and participative leadership styles [25]. Although nurses valued structured leadership, the moderate scores for consideration behavior suggest opportunities to strengthen the relational aspects of leadership. This could be related to the study context in which hierarchical systems may hinder relational leadership. Similar findings have been reported in Saudi Arabia, where nurses valued leaders who provided clear guidance while demonstrating emotional support, mutual understanding, and collaboration [26]. Similarly, a systematic review found that supportive and communicative leadership significantly improved job satisfaction and retention among nurses in Saudi Arabia [27]. International evidence has linked both initiating structure and consideration behaviors to improved employee performance although supportive leadership has a stronger effect on organizational commitment [28]. Overall, the present findings suggest that task‐oriented leadership is well established in Saudi hospitals, whereas supportive and relational leadership behaviors may require further development to enhance nurses’ motivation and job satisfaction.

The findings also indicate moderate job satisfaction among nurses. The key job satisfaction elements such as work conditions, including workload, staffing adequacy, recognition, compensation, and leadership support, were only partially satisfactory. The moderate overall score, therefore, reflects that nurses find meaning in their work but feel constrained by the structural conditions. This pattern aligns with Herzberg’s two‐factor theory, in which the motivational factors such as meaningful work and positive relationships increase satisfaction, whereas unmet hygienic factors such as compensation and working conditions reduce satisfaction [29]. These findings were consistent with a previous study conducted in Saudi Arabia. Alshaibani et al. (2024) reported that job satisfaction is a significant predictor of nurses’ intentions to remain in their positions [30]. Their findings are supported by Alilyyani et al. (2022) who reported that emotional support from leaders and positive team relationships contribute to professional stability among nurses [31]. Therefore, moderate levels of perceived leadership behavior play a critical role in promoting job satisfaction.

This study demonstrates a moderate positive relationship between leadership behavior and job satisfaction, which is consistent with studies by Jakobsson Larsson et al. (2024) and Kohnen et al. (2024), who reported that meaningful, well‐structured, and professionally supportive work environments and leadership enhance intrinsic motivation [32, 33]. Similarly, Haeckl and Rege (2024) emphasized that supportive leadership promotes employee engagement, organizational identification, and emotional well‐being, which contribute to higher job satisfaction [34]. Overall, the findings suggest that strengthening supportive and participative leadership behaviors may improve nurses’ job satisfaction and contribute to better retention outcomes by reducing turnover intention.

Turnover intention remained moderately consistent with the global evidence, indicating that nurse turnover remains a major workforce challenge. In a global meta‐analysis, Xu et al. reported an average turnover intention rate of 27.7% among critical care nurses, which emphasizes the impact of stressful working conditions [35]. Similarly, Galanis et al. found that workplace stress, burnout, and inadequate organizational support significantly increased nurses’ intention turnover intention [36]. The moderate turnover intention score in this study may have been influenced by the intrinsic and relational aspects of job satisfaction, which can buffer nurses’ turnover intention even when structural conditions fall short of expectations.

The multiple linear regression and mediation analyses demonstrated significant negative relationships between perceived leadership behavior and turnover intention. The indirect pathway through leadership behavior mediated 30.3% of the relationship between job satisfaction and turnover intention. To our knowledge, this study is among the first to examine perceived leadership behavior as a statistical mediator of the association between job satisfaction and turnover intention among nurses in Saudi Arabia. These findings imply that nurses who perceive leaders as structured, supportive, and relational are less likely to report turnover intention. These findings can be interpreted using the path–goal theory, which proposes that leaders who clarify roles, reduce barriers, and provide socio‐emotional support to their followers enhance employee satisfaction and reduce withdrawal behaviors [13]. This study also confirmed a well‐established negative correlation between job satisfaction and turnover intention. Recent studies similarly reported that dissatisfaction, burnout, and unmet expectations are strong predictors of turnover intention [37–39].

These findings highlight the relevance of leadership behavior in forming nurses’ experience and turnover intention. By testing this model among nurses in Saudi Arabia, where the healthcare workforce rely heavily on expatriate staff, it also provides evidence on how an established Western leadership framework operates within a difference cultural context, which highlights an important gap in the international leadership and retention research. However, other factors beyond leadership, such as structural factors, should be considered.

### 4.1. Implications

Healthcare organizations should consider leadership development programs that incorporate training in communication, conflict resolution, recognition strategies, and approaches to promote autonomy and trust in nursing teams. The mediating role of leadership behavior between job satisfaction and turnover intention highlights the importance of creating work environments that support psychological safety and professional development. Healthcare organizations should implement evidence‐based retention strategies, including equitable compensation schemes, adequate staffing, and effective reward systems. Investment in leadership development programs may help nurse managers strengthen their supportive, participative, and adaptive leadership competencies.

### 4.2. Limitations

First, the cross‐sectional design limits the causal relationships between the study variables. Although significant associations were identified, the direction of these relationships could not be confirmed. Therefore, longitudinal studies are recommended to examine the temporal relationships among these variables. Second, the use of self‐reported questionnaires might have caused response biases, such as social desirability and recall bias. Participants may have provided responses that they perceived as socially acceptable that did not reflect their actual experiences, particularly regarding leadership behavior and turnover intention. Third, the use of convenience sampling from a limited number of hospitals within one city may limit the generalizability. Differences among the selected hospitals, including organizational size and specialty, may also have influenced the results. Fourth, the findings reflect general direct‐care nurses and may not capture specialty‐specific dynamics (e.g., emergency or surgical units). Consequently, the findings may not fully represent the broader nursing workforce across all healthcare institutions in Saudi Arabia. Finally, leadership behavior was assessed based on nurses’ perceptions rather than direct observation, which may not accurately reflect actual leadership practices.

## 5. Conclusion

Among nurses employed at three hospitals in Riyadh, Saudi Arabia, lower job satisfaction and perceptions of leadership behavior were associated with higher turnover intention. Perceived leadership behavior partially mediated the relationship between job satisfaction and turnover intention. Strengthening supportive leadership behaviors through targeted leadership development may represent a practical, modifiable strategy to increase job satisfaction, reduce turnover intention, and mitigate nursing shortages.

## Funding

This work was supported by the Ongoing Research Funding Program of King Saud University (ORF‐2026‐856).

## Conflicts of Interest

The authors declare no conflicts of interest.

## Data Availability

The data that support the findings of this study are available from the corresponding author upon reasonable request.
